# Genomic profiles and tumor immune microenvironment of primary lung carcinoma and brain oligo-metastasis

**DOI:** 10.1038/s41419-021-03410-7

**Published:** 2021-01-21

**Authors:** Zhengbo Song, Ling Yang, Zhipeng Zhou, Pansong Li, Wenxian Wang, Guoping Cheng, Rongrong Chen, Lianpeng Chang, Yiping Zhang, Yanfang Guan, Xuefeng Xia, Xin Yi, Rongrong Zhou, Ming Chen

**Affiliations:** 1 Department of Clinical Trial, The Cancer Hospital of the University of Chinese Academy of Sciences (Zhejiang Cancer Hospital), Hangzhou, Zhejiang 310022 China; 2 Institute of Basic Medicine and Cancer, Chinese Academy of Sciences, Hangzhou, Zhejiang 310022 China; 3 Geneplus-Beijing Institute, Beijing, 102206 China; 4 Department of Pathology, The Cancer Hospital of the University of Chinese Academy of Sciences (Zhejiang Cancer Hospital), Hangzhou, Zhejiang 310022 China; 5 Department of Oncology, Xiangya Hospital, Central South University, Changsha, Hunan 410008 China; 6 Department of Radiotherapy, The Cancer Hospital of the University of Chinese Academy of Science (Zhejiang Cancer Hospital), Hangzhou, Zhejiang 310022 China

**Keywords:** Cancer microenvironment, Non-small-cell lung cancer, Metastasis, Tumour biomarkers, Tumour heterogeneity

## Abstract

Brain metastasis (BM) is a common malignant event in lung cancer. Here, we recruited 33 lung cancer patients with brain oligo-metastasis to explore the genomic features and tumor immune microenvironment (TIME) of the lung and BM independently. For genomic profiling, targeted sequencing was performed. We found that high-frequent *ZFHX3* occurred in the lung (40%) and brain tumor (28%), which might relate to brain metastasis event; the vast majority of patients had lesions-shared mutations in primary tumor and BM, confirming the common clonal events; and *EGFR* was the most frequently clonal gene in both lung and BM, indicating its driver capability. To characterize TIME status, we also sequenced the T cell receptor (TCR) repertoires and performed immunohistochemistry (IHC) on CD8+ tumor-infiltrating lymphocytes (TILs) and PD-L1 expression in 28 patients who had paired samples. Through the comparison, the TCR clonality of BM was higher than lung tumor, indicating the distinct pattern of the stronger oligoclonal T cell expansion in BM; the primary tumor had a higher TMB than oligo-BM (13.9 vs 8.7 mutations, *p* = 0.019); CD8 + TILs of BM were significantly lower than lung tumor (10% vs 30%, *p* = 0.015), revealing the lower level of cytotoxic T cell infiltration; BM showed statistically equivalent level of PD-L1 compared with lung tumor (*p* = 0.722). We further investigated the potential biomarkers associated with overall survival (OS) after brain surgery. We found that higher TCR clonality was related to prolonged OS in EGFR-treated patients (HR 0.175, *p* < 0.001) but the worse outcomes in non-EGFR-treated (HR 2.623, *p* = 0.034). More CD8+ TILs were an independently positive indicator for OS, in EGFR-treated (HR 0.160, *p* = 0.001) and non-EGFR-treated patients (HR 0.308, *p* = 0.009). These findings provide a meaningful molecular and clinical understanding of lung carcinoma and brain oligo-metastasis.

## Introduction

Lung cancer is the most common cause of cancer death worldwide. Brain metastases (BM) are detected at diagnosis in more than a quarter of patients with stage IV non-small-cell lung cancer (NSCLC)^1^. About 10% of NSCLC patients with early stage primary tumor receiving surgical resection are found to exhibit recurrence in the form of BM^2^. For SCLC, 10–21% are diagnosed with BM initially, and 50–80% will develop brain metastases during the course of the disease^3^. Although there are various treatments including surgery, stereotactic radiotherapy (SRT), whole-brain radiotherapy (WBRT), systemic therapy, and optimal supportive care^4^, more than half of patients with BM will die within a few months, indicating the poor prognosis^5^.

With the development of diagnosis and treatment technology, the outcomes of patients with BM were prolonged. Tyrosine kinase inhibitors (TKIs) for epidermal growth factor receptor (*EGFR*) mutation and anaplastic lymphoma kinase (*ALK*) rearrangements, targetable mutant genes commonly used to guide personalized targeted therapies for primary lung tumor, has shown high response rates for BM^6–8^. In addition, bevacizumab added to various chemotherapy agents or erlotinib in patients with NSCLC for BM showed safety and low incidence of CNS hemorrhage^9^. Immunotherapy with PD-1 or PD-L1 inhibitors is generally accepted treatment option for patients with advanced lung cancer, whether as a single agent or in combination with chemotherapy^10,11^. In the first-line KEYNOTE-189 trial^10^ and the second-line OAK trial^12^, patients with immunotherapy-treated BM in subgroup showed a longer overall survival (OS) than chemotherapy-treated. A recent phase 2 trial reported that pembrolizumab had activity in BM from NSCLC with PD-L1 expression at least 1% and was safe in selected patients with untreated BM^13^.

Characterization of the genomic and immune profiles is helpful to provide a better understanding basis for the precise treatment of BM. A recently published genomic characterization demonstrated clonally dominant and nearly universal genetic variations of BM and matched diverse primary tumor samples^14^. Genomic complexity and heterogeneity of primary tumor and matched BMs of Asian NSCLC were reported recently through target sequencing using a cancer-related gene panel^15^. The tumor immune microenvironment (TIME) has also been explored. Kudo et al.^16^ proposed that the TIME of BM was more immunosuppressed than primary tumor in western NSCLC using gene expression test and T-cell receptor (TCR) beta repertoire sequencing.

It is necessary to systematically assess the mutation and immune characteristics and investigate their relations of primary and BM, including mutations, clonal structure, antigen production, antigen recognition, lymphocyte infiltration, and immune checkpoint status, in more Asian samples. Here we collected 33 primary tumors and paired synchronous or metachronous BMs of lung cancer, performed a 1021-gene-panel (Supplementary Table 1) and TCR repertoires sequencing, and PD-L1 and CD8+ tumor-infiltrating lymphocytes (TILs) immunohistochemistry (IHC). Data were used to further compare the mutational differences, calculated the tumor mutation burden (TMB) and TCR clonality and diversity, estimated the clone structure, and showed expression of PD-L1 and the tumor-infiltrating CD8+ lymphocytes within subgroups. We found BM specific *ZFHX3* gene, higher TCR clonality and lower CD8+ TILs level in BM, and prolonged OS of lower BM TCR clonality group.

## Results

### Clinical characteristics of patients

Thirty-three patients with lung carcinoma were enrolled in this study, with median age of 58-year old (range 35–78) and with more male patients (73%) than female patients (27%). The majority of cases were with lung adenocarcinoma (LUAD, *n* = 21), followed by lung squamous cell carcinoma (LUSC, *n* = 6), small cell lung cancer (SCLC, *n* = 3), large cell carcinoma (LCC, *n* = 2) and lung neuroendocrine carcinoma (LNEC, *n* = 1). Patients with BM who underwent surgical resection of their primary or metastatic lesions either because of presentation with synchronous BMs (*n* = 16), or metachronous BMs were included (*n* = 17). Clinical characteristics and treatments of the 33 patients were summarized in Table 1.

**Table 1:** Clinical features of lung cancer patients with brain metastases.

Characteristics	All (*n* = 33)	Synchronous (*n* = 16, 48%)	Metachronous (*n* = 17, 52%)
Age (median, years)	57	61	56
≤55	13 (39%)	5 (31%)	8 (47%)
>55	20 (61%)	11 (69%)	9 (53%)
Gender			
Male	24 (73%)	13 (81%)	6 (35%)
Female	9 (27%)	3 (19%)	11 (65%)
Pathology			
LUAD	21 (64%)	10 (63%)	11 (65%)
LUSC	6 (18%)	3 (18%)	3 (18%)
SCLC	3 (9%)	2 (13%)	1 (6%)
LCC	2 (6%)	0 (0%)	2 (12%)
LNEC	1 (3%)	1 (6%)	0 (0%)

Of the 33 patients, 24 (72.7%) had both paired primary lung and BM samples available, 8 with only BMs and 1 with only lung samples available for 1021-gene-panel test. Twenty-eight paired samples were successfully performed TCR repertoires sequencing, CD8+ TILs, and PD-L1 expression to assess TIME status.

### Mutational characteristics of lung tumor and BM

**Fig. 1:**
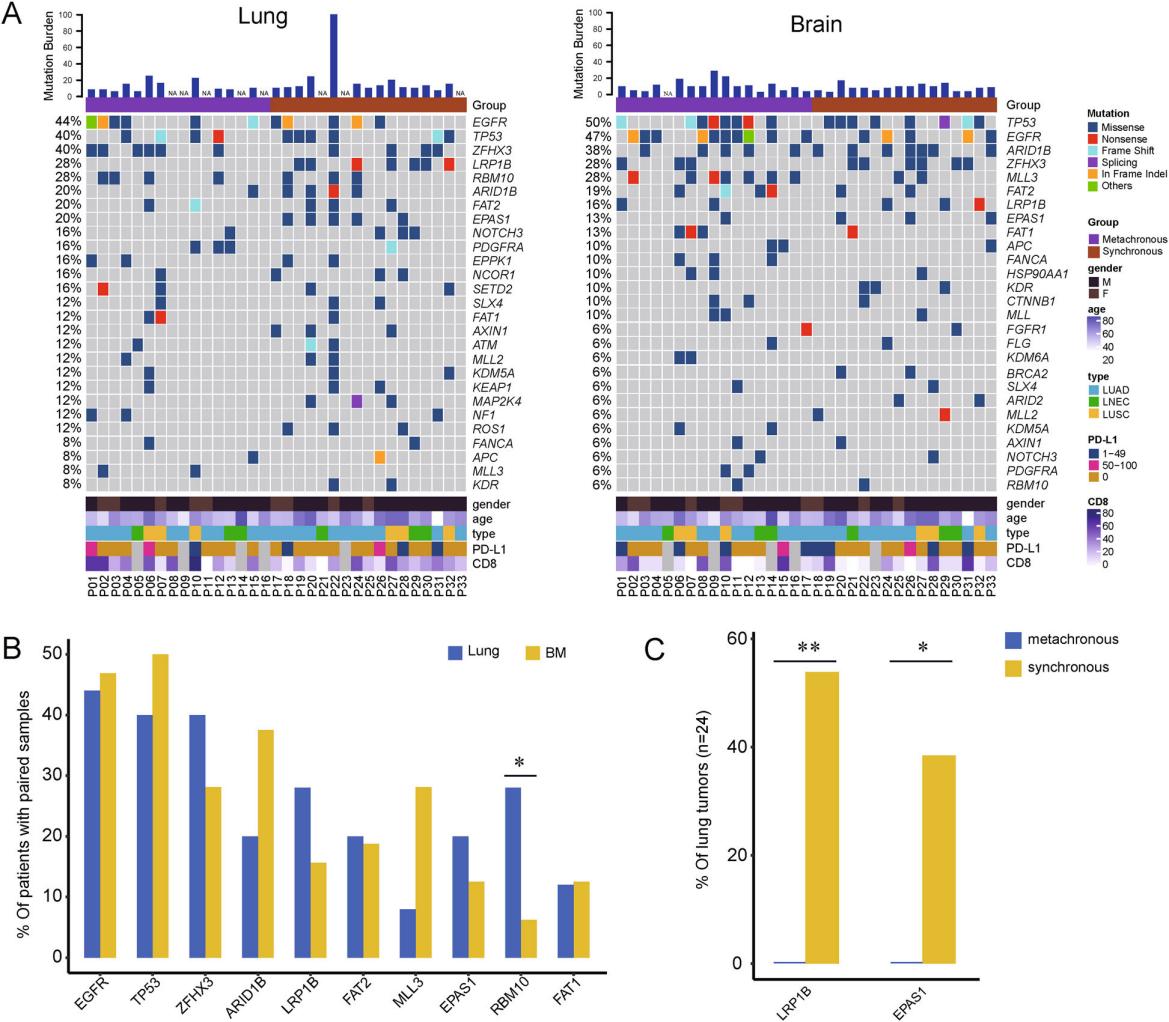
Mutational characteristics of primary lung and BM. **A** The mutational landscapes of primary and BM tumors. The not available samples for NGS test were marked as NA. **B** Comparison of top genes between primary and BM. **C** The different genes of lung tumors in synchronous and metachronous group. *p < 0.05, and **p < 0.01.

We examined the recurrently altered genes of the primary tumor and BM, respectively (Fig. 1A). *EGFR* (44%), *TP53* (40%), *ZFHX3* (40%), *LRP1B* (32%), and *RBM10* (28%) were the most frequent in primary tumors. *EGFR* and *TP53* mutations affected 71% (*n* = 17) of total. Of these, 4 had *EGFR*/*TP53* co-mutations, less than those with exclusive *EGFR* or *TP53*. Most (5 out of 7) of *RBM10* were co-mutated with *TP53*. In BM samples, the most common genes were *TP53* (50%), *EGFR* (47%), *ARID1B* (38%), *ZFHX3* (28%), and *MLL3* (28%). *EGFR* and *TP53* mutations affected 72% (*n* = 23) of patients. Of them, increased *EGFR*/*TP53* co-mutations (*n* = 8, 25%), although with no statistical difference compared to primary tumor.

We further assessed the essential differences of mutational features between primary tumor than BM, using the 24 patients with paired samples. *RBM10* was more frequent in primary tumor (Fisher’s exact test, *p* = 0.034). Overall, BMs showed genetic heterogeneity with slight differences of mutational prevalence compared with primary tumors (Fig. 1A, B). A comparison of mutational features between groups with synchronous and metachronous BMs was performed. *LRP1B* and *EPAS1* were found with a significantly higher incidence in lung tumors those with synchronous BM, than with non-synchronous (Fisher’s exact test, *p* = 0.005 and *p* = 0.039, respectively) (Fig. 1C). No different genes were found in synchronous and non-synchronous BMs.

### Clonal evolution of lung tumor and BM

**Fig. 2:**
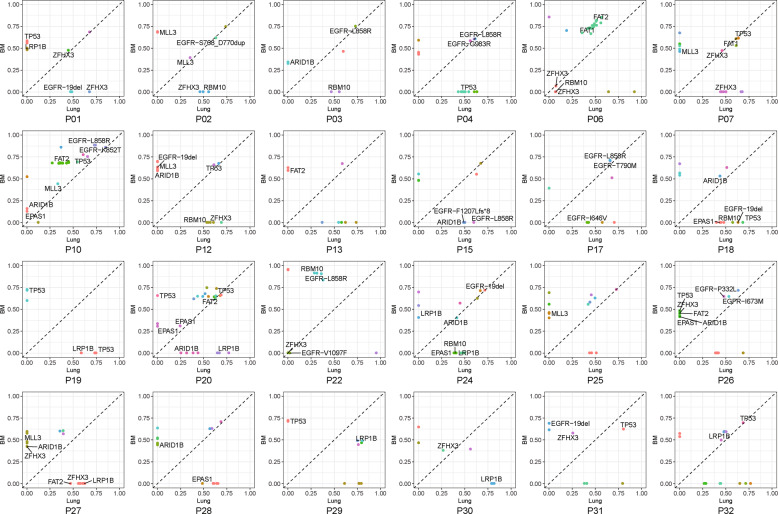
The mutational CCF plot of each patient with paired tumors. Each point color showed the same gene PyClone clusters in patients. The *X* and *Y* axis were cancer cellular fraction (CCF).

We investigated the clonal evolution of primary tumors and BMs. For all the 24 paired samples, 23 (96%) had at least one shared mutation (median 3, range 1–19), indicating the common genetic clonal events in primary tumor and BM. The only one without identified shared mutations was a LUAD patient. Nine (38%) had the shared mutations contained in the mutational cluster with the highest cancer cellular fraction (CCF), being considered as clonal, in both primary tumor and BM. Fourteen (58%) had shared mutations with inconsistent CCF in primary tumor or BM (Fig. 2). *EGFR* and *TP53* were the most frequently clonal genes in primary tumors, accounting for 16% of all patients and 75% of LUAD. In BMs, *EGFR* (22%) and *TP53* (16%) were similarly the most prevalent clonal genes. *RBM10*, *LRP1B*, and *EPAS1* mutations were all subclonal in lung tumors and BMs (Fig. 2).

### TCR characteristics of lung tumor and BM

**Fig. 3:**
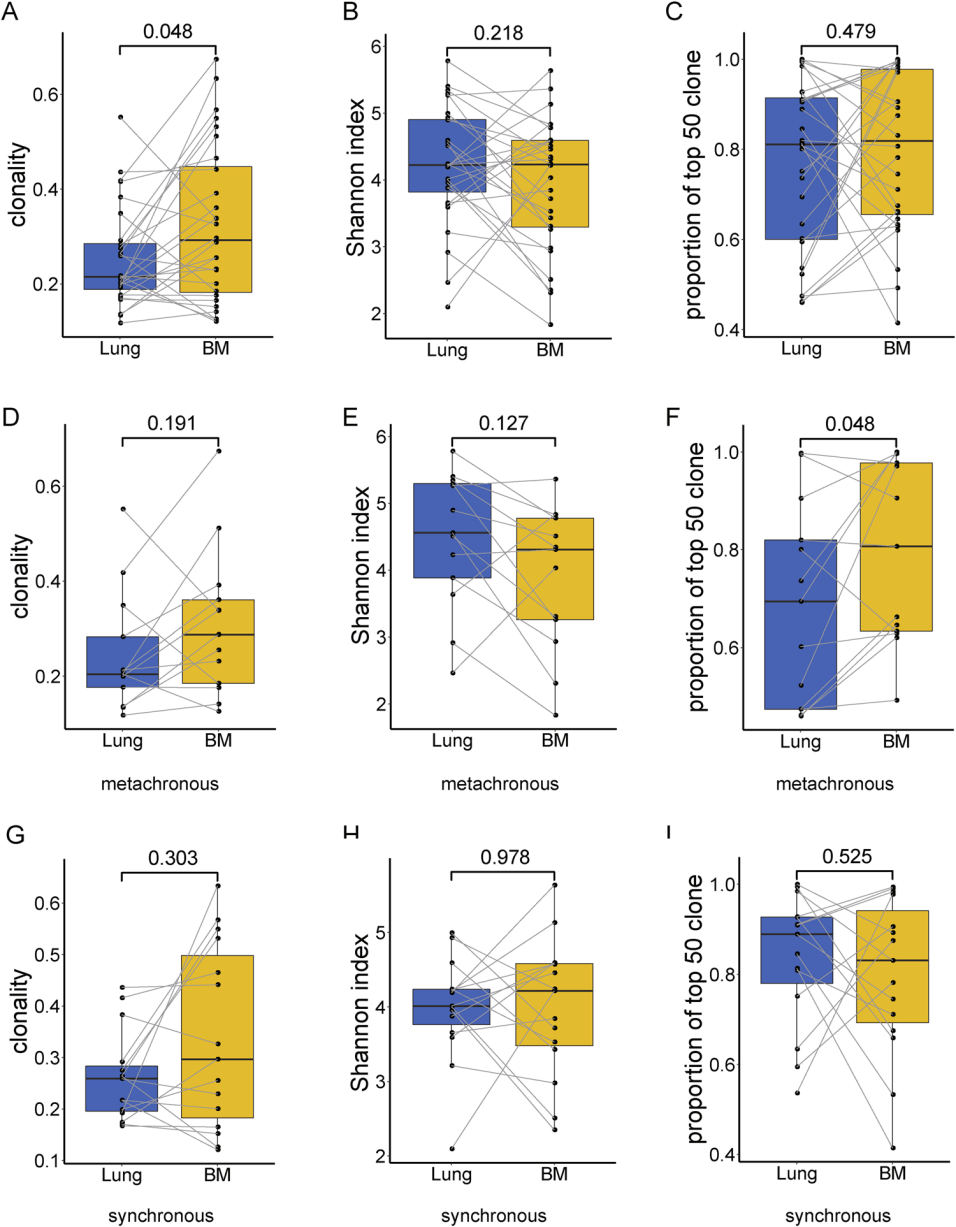
Higher clonality in BMs relative to primary lung tumors. Boxplot **A**, **B** and **C** shows the difference of lung and BM in clonality, Shannon index, and the frequency accumulation of the top 50 T cell clones, respectively. **D**, **E**, and **F** show the difference of clonality, Shannon index, and the frequency accumulation of the top 50 clones between lung and BM in the metachronous group. **G**, **H**, and **I** show the difference of clonality, Shannon index and the frequency accumulation of the top 50 clones between lung and BM in synchronous group. Statistical analysis was performed using the Wilcoxon paired test.

TCR clonality, Shannon index, and the top 50 clones in lung and brain tumors were compared and shared T cell clones between lung and brain tumors were analyzed. Clonality of BM was higher than paired primary tumor (Wilcoxon paired test, *p* = 0.048) (Fig. 3A), while no differences were showed in Shannon index or frequency accumulation of top 50 clones (*p* = 0.218 and *p* = 0.479, respectively) (Fig. 3B, C). We noted that more than half of the patients showed increased clonality (61%) and decreased Shannon index (61%) in BM than lung tumor (Fig. 3). A median of 228 (from 41 to 1107) and 348 (36 to 1951) unique T cell clones were identified in lung tumors and BMs, respectively. All patients had shared clones (median 25, range 2–281), and the majority (96%) had shared clones as part of the top 50 clones in both primary tumor and BM (Fig. S1). The median value of MOI (Morisita–Horn similarity index) between lung and brain tumors was 0.04 (2.98e−05 to 0.235).

We next investigated whether the metastatic type influenced the comparison of TCR indexes in primary and BM. TCR Clonality and Shannon index were with no significant differences between the lung and brain tumors in each subgroup of patients with BM synchronous or metachronous metastasis; metachronous BM had a marginally significant higher proportion of the top 50 clones than paired lung tumor (Fig. 3D–I). However, the noteworthy decreased *p* values in the metachronous group suggested the possibly different patterns of T cell expansion.

We found no relationship between TCR status (clonality, Shannon index, the top 50 clones, or MOI) and clinical features (age, gender, stage, pathology, and metastatic type), either in the lung or brain tumor. No relation between TCR status and *EGFR* mutation was found either in lung or brain tumor.

### TIME in lung and BM tumors

**Fig. 4:**
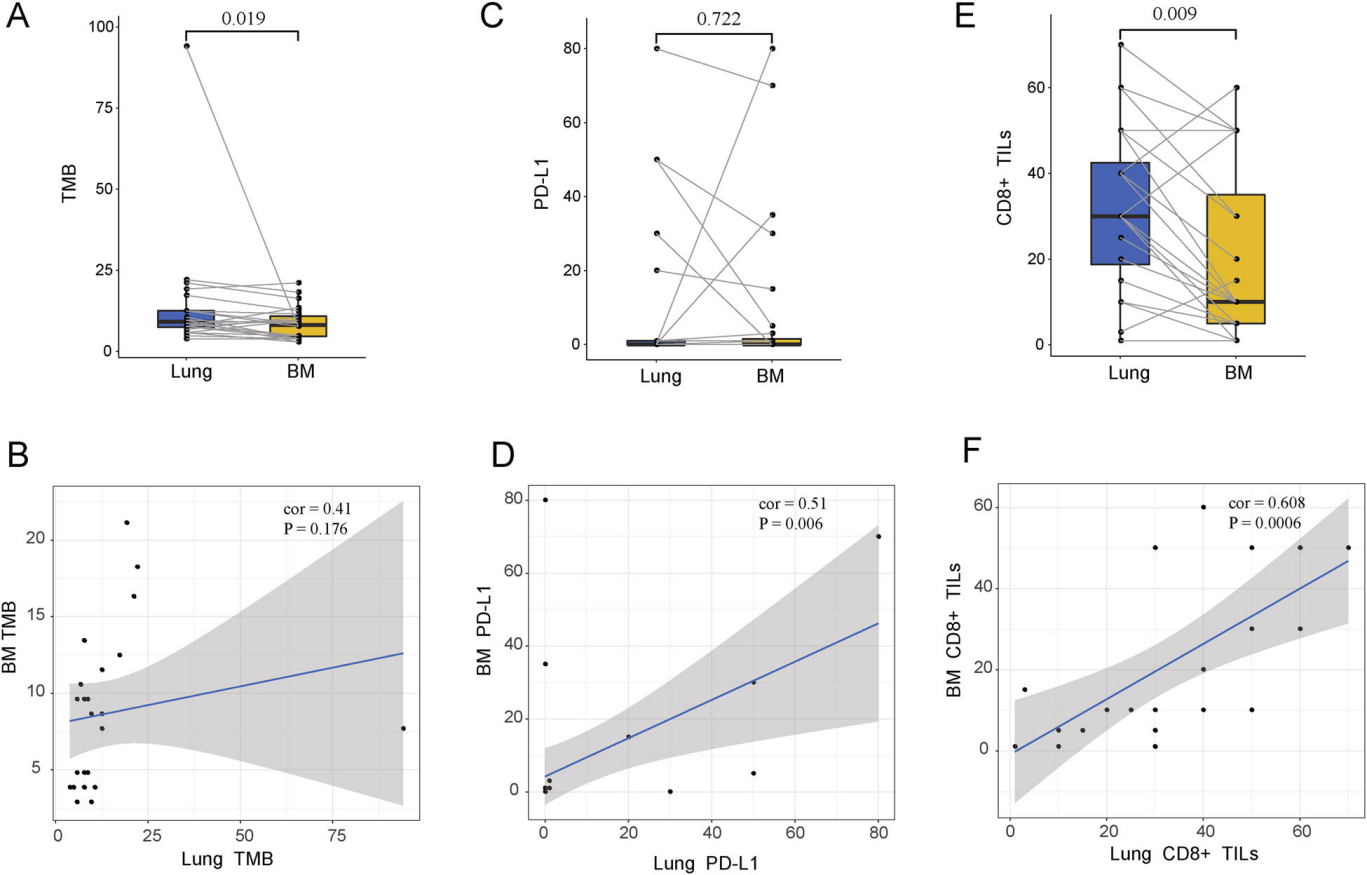
The differences and correlations in TMIE indexes between lung and BM. Comparison of TMB (**A**), PD-L1 (**C**) and CD8+ TILs (**E**) between lung and BM. **B**, **D**, **F** show the correlation between lung and BM in TMB, PD-L1, CD8+ TILs respectively. Statistical analysis was performed using the Wilcoxon paired test and Spearman correlation test.

We compared the TMB level, CD8+ TILs, and PD-L1 expression between lung and BM tumors to characterize the TIME. Primary tumor had higher TMB than oligometastatic BM (median 13.9 Vs 8.7 mutations, Wilcoxon paired test, *p* = 0.019), meanwhile there was no association between them, due to the only one hypermutated lung tumor (Spearman *r* = 0.41, *p* = 0.176) (Fig. 4A, B). Twenty-eight patients were performed PD-L1 expression and CD8+ TILs. Eight (28.5%) primary lung tumors and 10 BMs (35.7%) were PD-L1 positive (TPS > 0). Seven cases (85.7%) were PD-L1 bi-positive in lung and BM and 17 bi-negative cases (Fig. 1A). BM showed a statistically equivalent and proportional level of PD-L1 compared with lung tumor (median 0 vs 0, Wilcoxon paired test, *p* = 0.722, Spearman *r* = 0.51, *p* = 0.006) (Fig. 4C, D). CD8+ TILs of BM were significantly lower than lung tumor (median 10% vs 30%, Wilcoxon paired test, *p* = 0.009) and strongly proportional with BM (Spearman *r* = 0.61, *p* < 0.001) (Fig. 4E, F). These results indicated the TMIE heterogeneity and correlation of lung and brain tumor.

We next separated the synchronous and metachronous group to examine the TIME of lung and brain tumors (Fig. S2). In synchronous group, CD8+ TILs and PD-L1 expression were observed at similar levels between the lung and BM tumors, while lung tumors had a higher TMB (9.6 vs 7.7, Wilcoxon paired test, *p* = 0.039). In metachronous group, TMB and PD-L1 expression were equivalent respectively between lung and BM, while BM had a lower level of CD8+ TILs (Wilcoxon paired test, *p* = 0.015). We found there was no statistic differences in TMB, CD8+ TILs, and PD-L1 expression between synchronous and metachronous group of neither lung nor BM tumors. *EGFR* was found no relation with TMB, CD8+ TILs, or PD-L1 expression either in lung or brain tumor.

### Molecular factors affecting BM-operative outcome

**Fig. 5:**
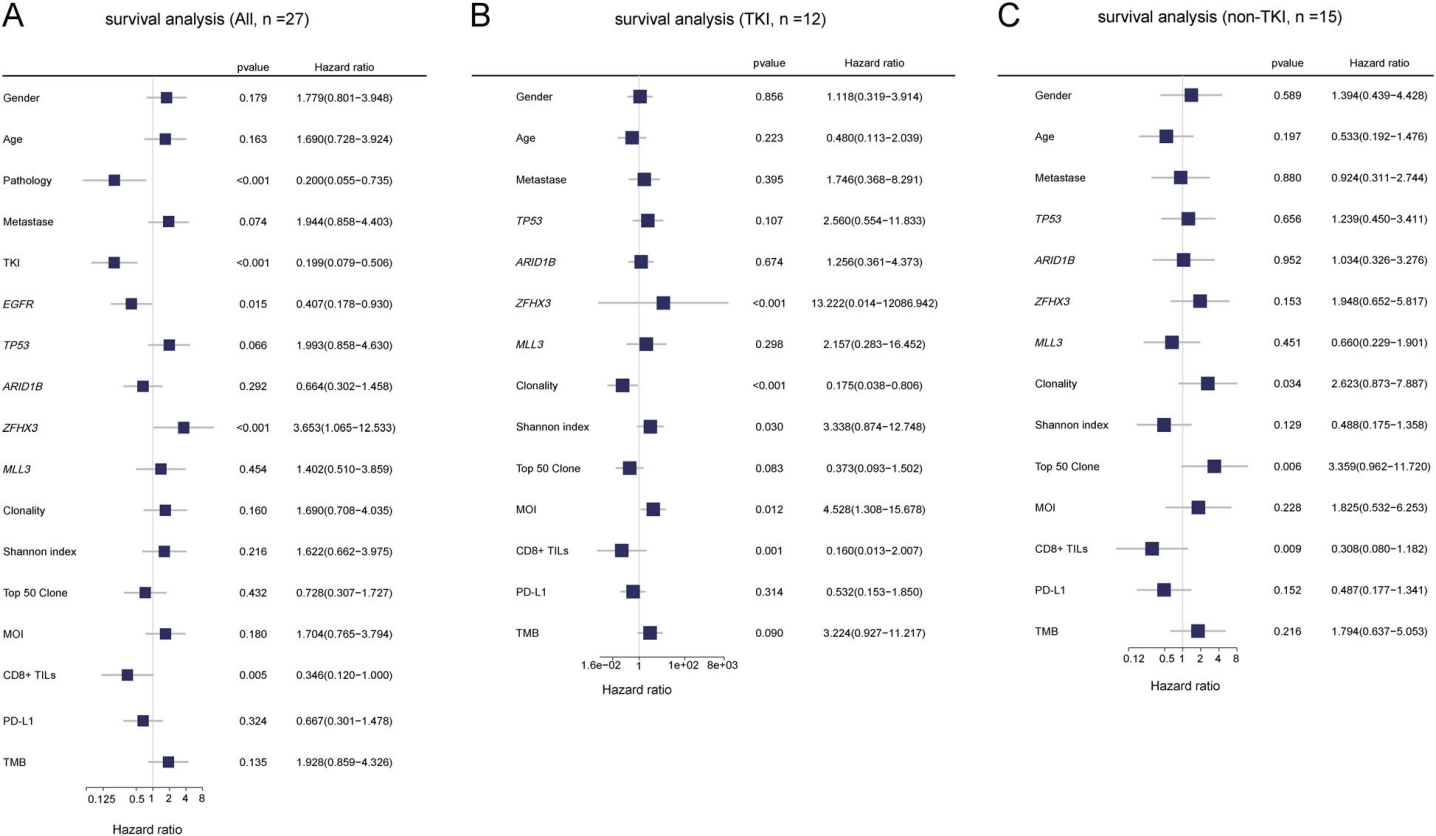
The analysis of molecular factor for prognosis. **A**, **B** and **C** show the result of survival analysis with all patients, patients receiving EGFR targeted therapy (TKI group), and no EGFR targeted therapy (non-TKI group). All molecular features fall into two categories, dichotomous and continuous: gender(male/female), pathology (LUAD/other), metastatic type (synchronous/metachronous), EGFR TKI therapy (with TKI/non-TKI), *EGFR* (positive/negative), *TP53* (positive/negative), *ARID1B* (positive/negative), *ZFHX3* (positive/negative), *MLL3* (positive/negative) and PD-L1 (positive/negative) were performed Kaplan-Meier survival analysis. For continuous factors, maximally selected rank statistics was performed to calculate optimal cutoffs. Those factors were divided into higher and lower groups according to cutoffs. Hazard ratio (HR) was calculated by high group compare to low group.

We scanned the impact of various clinical and molecular factors of BM on OS since the surgical operation of BM in this cohort. Pathology, BM-postoperative treatment, *EGFR*, *ZFHX3*, and CD8+ TILs level of BM have reported associations with OS (Fig. 5A). Considering the influences of the dominant pathological type of adenocarcinoma and most of the patients with *EGFR* mutations receiving EGFR-TKI targeted therapy after BM resection, we classified patients into TKI (*n* = 12, 100% LUAD) and non-TKI (*n* = 15) groups to further assess the clinical and molecular factors. Within TKI group, patients with *ZFH3X* mutation, higher Shannon index, and higher MOI between lung tumor and BM had poor outcome, while higher TCR clonality and more CD8+ TILs indicated prolonged OS and lower hazard ratio (Fig. 5B). Within non-TKI group, TCR clonality and CD8+ TILs were the only two indexes with statistical significance for patient stratification (Fig. 5C). We found that patients with higher TCR clonality showed as an indicator for worse outcome, which was the opposite of what was observed in the TKI group, while CD8+ TILs had a consistent trend.

## Discussion

BM is one of the most common metastases of lung cancer^17^, with a poor prognosis. In this study, we collected tumor samples of matched primary lung and brain metastases for comprehensive analysis of genomic profiles, TCR repertoires, CD8+ TILs, and PD-L1 expression. A range of lung cancer subtypes including SCLC and NSCLC enrolled in this cohort represented the pathological comprehensiveness for research. We have implemented meaningful explorations at the molecular and phenotypic levels and the interaction of them.

We examined the recurrent mutations and evolutionary genetic events in paired lung and brain tumors. Shared alterations confirmed the clonal relationship of them from the genomic level. Those clonal mutations with the highest CCF in both primary and brain tumors indicated the retention of dominance during the evolutionary process, while those mutations with inconsistent CCF suggested genetic selection events of tumor clone^18^. In fact, independent genetic events commonly occurred in the lung and brain, leading to genomic heterogeneity. Increased incidence of *RBM10* in lung and *ARID1B* and *MLL3* might be representatives. Higher expression of *RBM10* protein in tissue samples of lung cancer patients was reported associated with poor prognosis^19^. *ARID1B* was a member of the SWI/SNF chromatin remodeling complex that performs fundamental roles in gene regulation, cell lineage specification, and organismal development^20^. *MLL3*, a chromatin-regulating gene, was associated with improved survival in pancreatic cancer^21^. In addition, subclonal *LRP1B*^22^ and *EPAS1*^23^ enriched in lung tumor with synchronous BM indicated the relation of lung cancer with the risk of synchronous BM. Although we have observed no influences of these genes on survival in our cohort, it might not be ignored in large-scale researches.

It is worth noting that *ZFHX3* was one of lung and brain shared and relatively top-frequent genes in this cohort. *ZFHX3*, Zinc Finger Homeobox 3, was first identified as a suppressor of alpha-fetoprotein gene and is a tumor suppressor in several cancers^24,25^. *ZFHX3* was enriched in lung cancer and breast cancer with BM compared with no metastasis in MSK-IMPACT data (https://www.cbioportal.org/)^26^. Minamiya et al. reported that suppression of *ZFHX3* expression in tumor cells could decrease the survival rate among patients (*n* = 140) with NSCLC^27^. Correspondingly, in our cohort, LUADs carrying the *ZFHX3* mutation and receiving EGFR-TKI had a higher risk of death. These results provided pieces of evidence of the relation of *ZFHX3* and BM and worse outcome of mutant patients.

We depicted the different and correlative TIME features of brain tumor compared to lung carcinoma. In total population here, a lower abundance of CD8+ TILs and higher TCR clonality in BM than lung tumor indicated that, despite the difficulty of being infiltrated by blood-transporting lymphocytes due to the blood-brain barrier (BBB), there was the possibility of oligoclonal T cell expansion in BM. Effective T cell response involves activation and expansion of specific antigen-reactive T cell clones^28^. Studies reported that increased T cell clonality was associated with anti-PD1 response in immunotherapy-naive patients with melanoma^29^ and a more clonal T cell repertoire was predictive of response to PD-1 blockade in melanomas^30^. In addition, equivalent PD-L1 levels compared to lung tumor provide the brain lesion molecular basis for receiving treatments using immune checkpoint inhibitors (ICIs), although depending on the emergence of drugs that could break through BBB. These results and researches suggest us for patients who have biomarkers supporting the use of ICI both in the lung and brain tumors, after the brain tumor is removed, systemic treatment using ICIs possibly provides benefit.

Surgical resection for an oligometastatic brain tumor is one candidate of treatments^31^. Surgical removal of a BM can lead to immediate elimination of life-threatening or symptom-generating mass effect and elimination of the source of perifocal edema. Molecular markers for the prognosis of brain surgery were still unmet. In our cohort, higher CD8+ TILs level in brain tumor had a prolonged OS for either *EGFR*-mutant LUADs who receiving TKI treatments or patients with lung cancer who not receiving TKI treatments, indicating CD8+ TILs level as a robust prognostic factor. We found that higher TCR clonality of BM in *EGFR*-mutant LUADs who receiving TKI treatments but lower TCR clonality in patients with lung cancer who not receiving TKI treatments had a prolonged OS since brain tumor resection. This indicated that specific T cell expansion in brain tumor might play a positive role in TKI response in *EGFR*-mutant LUAD, but a negative role in other conditions. A limitation is that the small sample size due to the unavailability of BM. Thus, large-scale research will be needed to further confirm the observation.

In summary, *ZFHX3* might relate to BM in lung cancer. Lower abundance of CD8+ TILs and higher TCR clonality in BM than lung tumors were characterized. BM *ZFHX3* mutation, CD8+ TILs, and TCR clonality level were potential factors of prognostic stratification for the surgery of oligometastatic brain tumor.

## Materials and methods

### Patients and tumor samples

Advanced lung cancer patients with oligometastatic BM were diagnosed and enrolled at Zhejiang Cancer Hospital from Sep 2008 to Oct 2017. All patients were indeed treatment-naïve. Biopsy and resected primary lung tumor and resected BM were collected. All tissues were reviewed through histopathological assessment by two board-certified pathologists and only samples contained ≥20% tumor percentage were performed NGS test. The study was approved by the Ethics Committees of Zhejiang Cancer Hospital. All patients had signed the informed consent form.

### DNA extraction

Genomic DNA from FFPE tumor samples was isolated using QIAamp DNA FFPE Kit (Qiagen, Hilden, Germany) according to the manufacturer’s protocol. 2100 Bioanalyzer (Agilent, Santa Clara, USA) spectra and quantification of DNA were used for quality control. DNA samples were fragmented to ~200 bp peak size by sonication before library construction.

### Next-generation panel sequencing and bioinformatics analysis

Indexed sequencing libraries were prepared using the protocols recommended by the Illumina TruSeq DNA Library Preparation Kit (Illumina, San Diego, CA). Sequencing libraries were hybridized to custom-designed probes (NimbleGen, Roche) of biotinylated oligonucleotides. Massively parallel sequencing was performed using an Illumina HiSeq 3000 (Illumina). Reads were aligned to the reference sequence b37 edition from the Human Genome Reference Consortium using BWA version 0.5.9 (Broad Institute). Hybridization capture sequencing revealed a mean effective depth of coverage over 1000× in both the lung and BM samples. Single nucleotide variants (SNVs) were called using MuTect (version 1.1.4) and NChot^32,33^. Small insertions and deletions (Indels) were determined by GATK^34^. TMB was interrogated using the number of nonsynonymous SNVs and Indels with the variated allele frequency (VAF) ≥ 1%.

### TCRβ sequencing

Immunosequencing of the human TCRβ CDR3 (third complementarity-determining region) was performed using the IR-seq platform from Geneplus-Beijing^35^. DNA from FFPE was amplified in a bias-controlled multiplex PCR system, followed by high-throughput sequencing. T-cell clonality was defined as 1-Peilou’s evenness and was calculated on productive rearrangements by:

$${\mathrm{Clonality}} = 1 - \frac{{\mathop {\sum }\nolimits_{i = 1}^R p_iInp_i}}{{InN}}$$

where _*pi*_ is the proportional abundance of rearrangement *i*, and *N* is the total number of rearrangements. MOI is a measure of the similarity in the T-cell repertoire between samples ranging from 0 to 1, taking into account the specific rearrangements and their respective frequencies, with a MOI of 1 being an identical T-cell repertoire^36^.

### IHC for CD8 and PD-L1

IHC for CD8 and PD-L1was performed as we have done previously^37^. Briefly, Immunohistochemical analysis was performed using primary antibodies against PD-L1 (SP263, 1:2000, RocheVENTANA, Tucson, AZ, USA). CD8+ TILs were detected by monoclonal antibody (4B11, RTU, Leica Biosystems, Buffalo Grove, IL, USA) using an automated stainer (BOND RX, Leica Microsystems). The immunohistochemistry results were based on the degree and intensity of cell membrane staining, with a tumor proportion score (TPS).

### Statistical comparisons

Mann-Whitney test was used in different groups carrying unpaired data. The Fisher’s exact test was used to compare proportions between two groups. Wilcoxon paired test was used for comparison of paired data. Spearman correlation analyses were performed using R software. OS was followed from the time of resection of the brain metastases to death. Kaplan–Meier survival analysis was used to compare OS. For numerical variables, we used the maximally selected rank statistics in survminer package to determine the optimal cutoffs. Two-tailed *p* < 0.05 were considered statistically significant.

## Supplementary information

Supplementary Table 1. Detailed List of the 1021-gene-panel.

Figure S1

Figure S2
