# Supplementary material for: Genomic profiles and tumor immune microenvironment of primary lung carcinoma and brain oligo-metastasis
Source: Cell Death Dis. 2021 Jan 21;12(1):106. doi: 10.1038/s41419-021-03410-7 (PMC7820277; doi:10.1038/s41419-021-03410-7)
Supplement: Supplementary file 1 — Supplementary Table 1. Detailed List of the 1021-gene-panel. [file 41419_2021_3410_MOESM1_ESM.docx]

**Supplementary Table 1. Detailed List of the 1021-gene-panel.**

ABCA10 ABCA8 ABCB7 ABCC8 ABCF2 ABL1 ABL2 ACE ACER2 ACOT11 ACPP ACSL1 ACSM5 ACSS3 ACTL6B ADAM23 ADAM33 ADAMTS12 ADAMTS16 ADAMTS19 ADAMTS20 ADAMTS5 ADAMTSL1 ADD2 AGMAT AGTPBP1 AHCTF1 AK5 AKR1B10 AKR1C1 AKT1 AKT2 AKT3 ALDH1A3 ALDH2 ALG5 ALK ALX4 AMOT ANK2 ANKRD13D ANKRD20A4 ANKRD27 ANKRD28 ANKRD30A ANKRD30B ANKRD36B ANO2 AP1B1 AP1G2 AP3B1 APAF1 APC APLP2 APMAP APPL2 AQP12A AR ARAF ARFGAP1 ARFRP1 ARHGAP35 ARHGAP40 ARHGEF1 ARHGEF7 ARNTL ARPC4-TTLL3 ASH2L ASTN1 ASXL2 ATAD2B ATG9B ATM ATP10B ATP10D ATP12A ATP2C1 ATP6V0A2 ATP8B2 ATR ATXN2 ATXN7L2 AURKA AURKB AXL BAP1 BAX BBS9 BCAS1 BCAS2 BCL2 BCL2L11 BCR BLOC1S1 BMPR1B BRAF BRCA1 BRCA2 BRD2 BRD3 BRD4 BRF1 BRSK2 BRWD3 BSG BTK BTNL3 BTRC C11orf30 C12orf5 C19orf38 C1orf112 C1orf35 C1QA C1S C20orf112 C2orf47 C2orf62 C7orf53 C9orf114 C9orf43 CACNA1A CACNA1D CACNA1E CADM2 CAMKK1 CAPRIN1 CARS CARS2 CASC4 CASP8 CASP8AP2 CASQ2 CATSPER2 CBFB CBL CBX4 CCDC155 CCDC159 CCDC17 CCND1 CCND2 CCND3 CCNE1 CCT3 CCT6B CD1E CD274 CD300LF CD5L CD9 CD97 CD99 CDH1 CDH18 CDH24 CDH26 CDK11A CDK12 CDK13 CDK14 CDK18 CDK19 CDK4 CDK6 CDK8 CDKN1A CDKN1B CDKN2A CDKN2B CDS1 CEACAM20 CECR2 CELA2B CGN CHD3 CHD4 CHD6 CHEK1 CHEK2 CHI3L1 CISD3 CLCN7 CLEC16A CLINT1 CNGB3 CNKSR2 CNOT3 CNOT4 CNTN1 CNTN4 CNTN5 CNTNAP3B CNTNAP5 COASY COL14A1 COL16A1 COL19A1 COL1A1 COL25A1 COL4A5 COL4A6 COL5A1 COL5A2 COL5A3 COL6A5 COL6A6 COL9A1 COPA COPG1 CPA1 CPSF3 CPSF6 CRKL CRTAM CRTAP CRYBG3 CSF1R CSMD1 CSMD3 CSN3 CSNK1E CSPP1 CTCF CTIF CTNNA2 CTNNB1 CTSF CYP2A13 CYP3A4 CYP4A11 CYTH4 DCLK2 DCST1 DDB1 DDR1 DDR2 DDX24 DDX3X DEPDC4 DGKK DHCR24 DHDDS DHX9 DIAPH1 DKC1 DLST DMD DMXL1 DMXL2 DNAH10 DNAH5 DNAH9 DNAJC11 DNAJC9 DNMT3A DNTTIP1 DOCK11 DOCK3 DOT1L DPP10 DPP4 DRGX DUOX1 DYSF DZANK1 ECHDC1 EDN1 EEF1A1 EFCAB5 EFCAB6 EFCAB7 EFHA2 EFNA5 EGFR EIF1AX EIF2B5 EIF2C2 EIF3E EIF3I EIF4ENIF1 EIF4H ELAVL3 ELL3 EMID2 ENPP2 ENTPD6 EPB41L2 EPB41L4B EPHA2 EPHA3 EPHA5 EPHB1 EPS8L3 ERBB2 ERBB3 ERBB4 ERCC1 ERG ESD ESR1 ETNK2 ETV6 EXOC4 EXOC5 EXOC6 EXOC7 EXTL3 EYA4 EZH2 F8 F9 FAH FAM114A2 FAM131B FAM135B FAM13C FAM157B FAM177B FAM21A FAM3A FAM49A FAM49B FAM5C FAM86B1 FAN1 FANCC FASTK FAT1 FATE1 FBN2 FBXW7 FCGR2A FCGR2B FCGR3A FDCSP FGFR1 FGFR2 FGFR3 FGFR4 FLCN FLNC FLOT2 FLT1 FLT3 FLT3LG FLT4 FMN2 FMNL3 FNDC4 FNIP2 FOLH1 FOXA1 FOXJ2 FOXL2 FRG1 FRG2B FRMD4A FRMPD2 FRMPD4 FSD2 FSHR FUBP1 FUNDC1 GAB2 GAB3 GABRD GAD2 GALNT13 GALNT14 GATA3 GFRAL GIGYF1 GINS4 GIPR GKN2 GLB1L3 GLYR1 GMDS GNA11 GNAQ GNAS GNPTAB GOLGA4 GPAT2 GPATCH2 GPR114 GPR125 GPR133 GPR144 GPS2 GRIA3 GRIK2 GUCY1A3 GUCY2C GYLTL1B HAAO HAP1 HAUS5 HAUS6 HCN1 HDAC1 HDAC4 HDAC6 HEATR7B2 HECTD4 HECW1 HECW2 HGF HID1 HIST1H3B HMCN1 HMHA1 HNF4A HOMER2 HPS3 HPS4 HRAS HSPA12B HSPD1 HYDIN IBSP IDH1 IDH2 IFT172 IGF1R IGSF9 IKBKAP IKBKE IL11RA IL13RA2 IL1RAPL1 IL27RA IL7R IMPG1 INHBA INPP4B INPP5J IQCA1 IRS2 ITFG2 ITGA8 ITGA9 ITIH1 ITLN2 ITM2A ITPKB ITPR1 JAK1 JAK2 JAK3 KCNAB2 KCNH6 KCNQ2 KDM4A KDM6A KDR KEAP1 KIAA0195 KIAA0226 KIAA0319 KIAA0922 KIAA1191 KIAA1199 KIAA1211L KIF13A KIF1B KIF26B KIF5B KIFAP3 KIFC1 KIR2DL3 KIR3DL3 KIT KLHL1 KLHL14 KLK1 KRAS KRT2 KRT9 KRTAP5-5 KTN1 L3MBTL1 LARP1 LCN10 LCT LCTL LETM1 LGALS13 LILRB3 LILRB4 LIPN LMAN1L LMBR1L LPCAT4 LPHN3 LRBA LRP1B LRP2 LRP4 LRRC16B LRRC2 LRRC7 LRRC72 LRRD1 LRRFIP2 LRSAM1 LTBP1 LUC7L2 LUZP4 MAEL MAGI1 MAN2A1 MAP2 MAP2K1 MAP2K2 MAP2K4 MAP3K1 MAP4K1 MAPK1 MAPK3 MAPKAPK3 MAPRE3 MAST1 MBIP MBTPS2 MCF2L2 MCL1 MCOLN2 MDGA2 MDM2 MDM4 MDN1 MED12 MED23 MEFV MET METTL14 METTL5 MGAM MICALL1 MID1 MIER2 MITF MLH1 MLH3 MLL MLL3 MLL4 MLPH MORC1 MORN1 MPL MRPL1 MRPL24 MRPS18B MS4A1 MSH2 MSH3 MSH6 MSI1 MTA2 MTM1 MTOR MTR MTTP MUC5B MUS81 MYB MYBPC2 MYC MYCBP2 MYD88 MYH15 MYH2 MYH4 MYH8 MYH9 MYL5 MYL6 MYLK2 MYO3A MYOM1 NACAD NARF NAT10 NAV3 NBPF1 NBPF10 NCF2 NCKAP1 NCOR1 NCOR2 NEK5 NELL1 NF1 NF2 NFE2L2 NIPBL NLGN3 NLRC3 NLRP4 NMI NOP2 NOS1 NOS2 NOTCH1 NOTCH2 NOTCH3 NOTCH4 NRAS NRXN1 NRXN2 NT5C3L NTM NTRK1 NTRK3 NUDCD2 NUP205 NUP210 NUTM1 NWD1 NXF1 NXF5 OBP2A OBP2B OCA2 ODZ3 OR2T4 OR4A15 OR4C6 OR5L2 OR6F1 OSBPL10 OTOA OTOGL OVCH1 P4HB PABPC4 PACS2 PAEP PAGE1 PALB2 PARK2 PARP4 PCK2 PCLO PCNT PCNXL2 PCSK5 PCYT1A PDCD6 PDE1C PDE2A PDE4DIP PDGFRA PDGFRB PDIA5 PDILT PDK1 PDRG1 PEX6 PGAP1 PHACTR3 PHF20L1 PHYH PI4KB PIK3CA PIK3CB PIK3R1 PIK3R2 PIP4K2C PIP5K1C PIWIL1 PKD1L2 PKHD1 PKLR PLAC8 PLCB4 PLCZ1 PLEC PLK2 PLOD3 PLXNA1 PMS1 PMS2 POLDIP2 POLE POLR2J POLR3B POLR3GL POLRMT POM121L12 POTEG PPA1 PPDPF PPEF1 PPFIBP2 PPIL2 PPP1R17 PPP4R4 PQBP1 PREB PREX2 PRKAA1 PRKACA PRKAG3 PRKCD PRKDC PRKX PRRX1 PRSS1 PRUNE PSG2 PSG5 PSIP1 PSMB1 PSMB5 PSMC4 PSMC6 PSTPIP1 PTBP3 PTCD3 PTCH1 PTCH2 PTEN PTGES3L-AARSD1 PTGS2 PTPLAD1 PTPN11 PTPN13 PTPRA PTPRD PTPRM PYHIN1 QRICH2 RAB1B RAB3GAP2 RAB6A RAC2 RAF1 RALBP1 RAPGEF2 RARA RARB RASEF RB1 RBM6 RBMX RCC1 REC8 REG1B RELN RERE RET RFWD2 RFX3 RHEB RHOA RICTOR RNF215 RNF219 RNF43 ROCK1 ROS1 RPL22 RPL36A RPS5 RPS6KA1 RPS6KB1 RPTOR RPUSD4 RREB1 RRP7A RUNDC3A RUNX1 RYR2 RYR3 SAFB2 SAG SAGE1 SAMD8 SCN10A SCN3A SCN7A SCN9A SDK2 SEC14L4 SEC24B SEH1L SELP SEMA6A SEPT12. SERPINA7 SETD1B SETD2 SF1 SF3B1 SF3B14 SF3B3 SGCZ SGIP1 SGK1 SGPL1 SH2D3A SH3BGR SH3PXD2A SHISA4 SI SIDT2 SIK3 SIM1 SIM2 SLC13A3 SLC17A6 SLC17A8 SLC25A1 SLC25A30 SLC26A3 SLC2A2 SLC30A5 SLC35B2 SLC35B4 SLC38A4 SLC38A5 SLC43A1 SLC45A1 SLC4A10 SLC4A4 SLC5A1 SLC6A5 SLC8A1 SLCO1B7 SLCO5A1 SMARCA4 SMARCB1 SMO SMTN SNTG1 SORCS3 SPAG16 SPATA13 SPG20 SPINT1 SPPL2A SPPL3 SPRED1 SPTA1 SRC SRRT SSBP3 SSH2 SSPO ST18 ST6GALNAC1 STAG2 STAT1 STAT3 STAT4 STAT6 STK11 STK11IP STK31 STX3 SULT1A4 SUPT5H SUPT6H SYCP2L SYK SYNE1 SYNE2 SYNJ2 TAF1B TAF6 TARBP1 TBC1D1 TBC1D21 TBC1D3 TBC1D5 TBL1X TBP TBX15 TBX22 TBX3 TCF20 TCF4 TCP10 TCP11 TEK TERT TESC TEX35 TFDP1 TGDS TGM2 TGM5 THBS2 THEM5 THOC1 THSD7A THSD7B TIMD4 TIMM44 TIMP3 TJP3 TLE1 TLL1 TMC2 TMED8 TMEM104 TMEM120B TMEM132D TMEM145 TMEM247 TMEM80 TMEM87A TMPRSS2 TMTC4 TMX3 TNFAIP6 TNFSF4 TNN TNNT1 TNR TNS3 TOP1 TP53 TP53BP1 TPCN1 TPH2 TPMT TPTE TRIM33 TRIM51 TRIM58 TRIML1 TRIO TRIP11 TRMT112 TRPC5 TRUB1 TSC1 TSC2 TSGA10 TSKS TSPAN12 TSR2 TTF2 TTN TUBA3C TUBGCP4 TUBGCP5 TYK2 TYRP1 U2AF1 U2AF2 UBASH3A UBE2Q1 UBE4B UCHL3 UCK2 UGT8 ULK3 UMOD UNC13A UNC13D UNC5D USP12 USP34 USP39 USP45 USP48 VAV1 VEGFA VEZF1 VHL VILL VIT VPS13A VPS33B VSIG4 WAS WASL WDR44 WDR52 WDR62 WDR66 WDR72 WDTC1 WLS WSCD2 WWP2 XBP1 XPO1 XPO4 XPO5 XRCC1 ZAP70 ZBTB8OS ZC3H13 ZC3H7B ZDHHC11 ZFC3H1 ZFR ZMYM4 ZNF143 ZNF350 ZNF385A ZNF414 ZNF512B ZNF541 ZNF563 ZNF614 ZNF687 ZNF705B ZNF705G ZNF711 ZNF804B ZSWIM8
