# Supplementary figures and images for: Genomic profiles and tumor immune microenvironment of primary lung carcinoma and brain oligo-metastasis
Source: Cell Death Dis. 2021 Jan 21;12(1):106. doi: 10.1038/s41419-021-03410-7 (PMC7820277; doi:10.1038/s41419-021-03410-7)

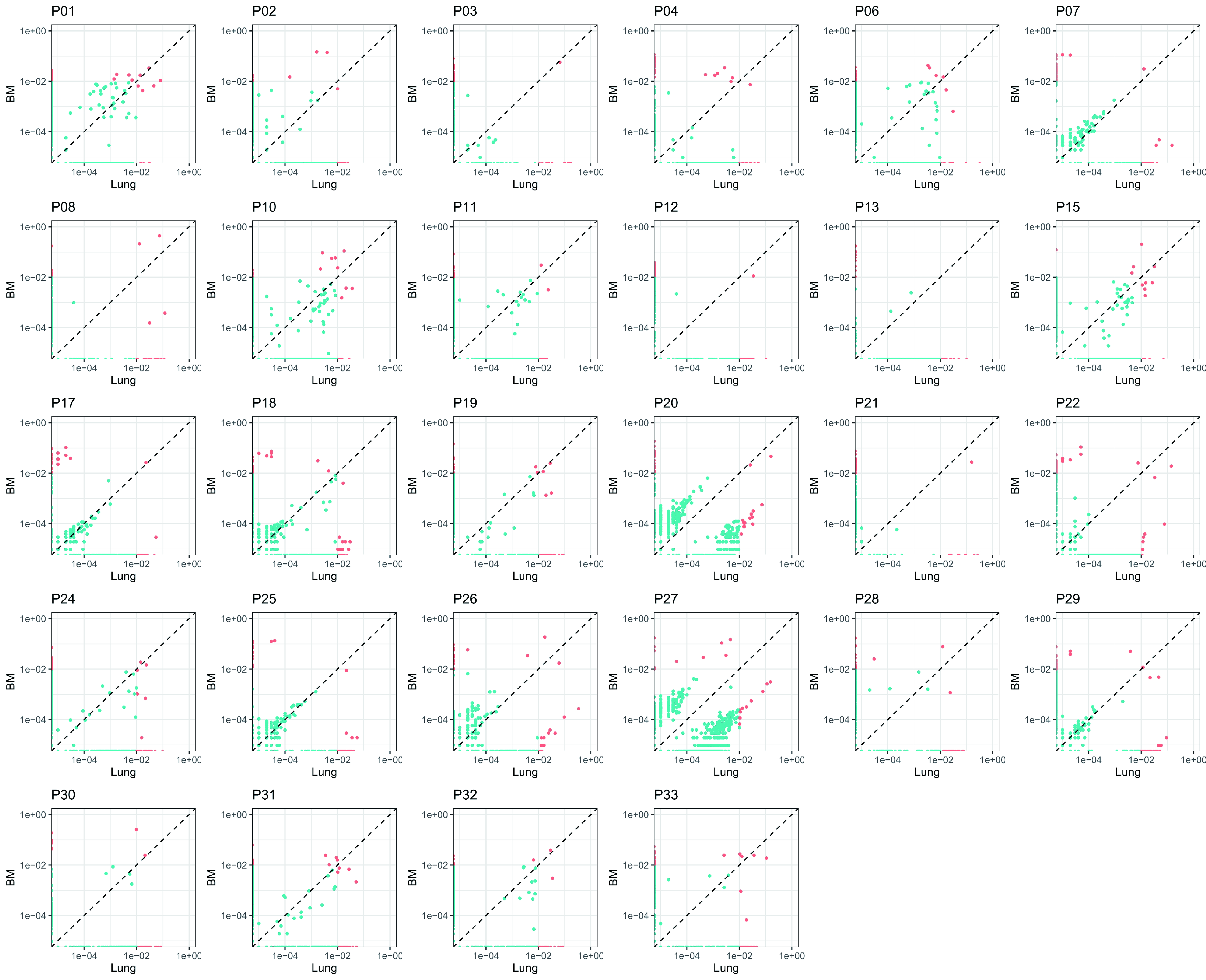

Supplement: Supplementary file 2 — Figure S1 [file 41419_2021_3410_MOESM2_ESM.tif]

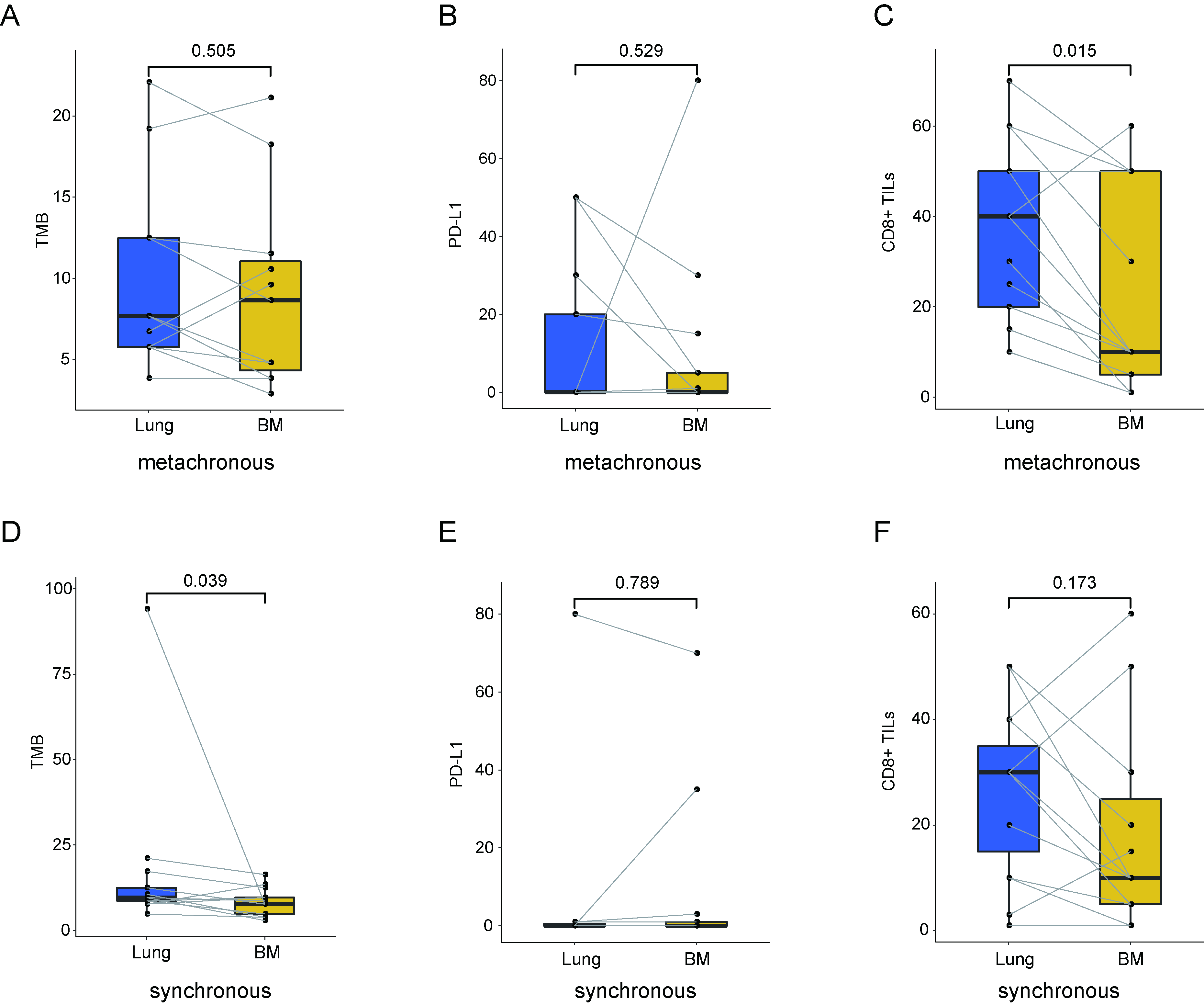

Supplement: Supplementary file 3 — Figure S2 [file 41419_2021_3410_MOESM3_ESM.tif]
